# Clinical and genetic characteristics of Cornelia de Lange syndrome in pediatric patients

**DOI:** 10.1002/ped4.70013

**Published:** 2025-07-02

**Authors:** Xiaoqiao Li, Ming Cheng, Min Liu, Wenjing Li, Yuchuan Li, Bingyan Cao, Liya Wei, Yuan Ding, Xi Meng, Lele Li, Chunxiu Gong

**Affiliations:** ^1^ Department of Endocrinology, Genetics, Metabolism Beijing Children's Hospital Capital Medical University, National Center for Children's Health Beijing China

**Keywords:** Cornelia de Lange syndrome, Genotype, Growth hormone, Phenotype

## Abstract

**Importance:**

Cornelia de Lange syndrome (CdLS) is a rare genetic disorder characterized by a spectrum of developmental and physical anomalies. Understanding the clinical and genetic landscape of CdLS in pediatric patients is crucial for improving diagnosis and management.

**Objective:**

To investigate the clinical and genetic characteristics of 19 pediatric patients with CdLS in China, with a focus on identifying the association between genetic variants and clinical severity.

**Methods:**

We performed whole exome sequencing on 19 patients with CdLS and compared their clinical characteristics based on the presence of null variants.

**Results:**

Among the 19 patients, 16 (84.2%) showed global developmental delays and 14 (73.7%) experienced prenatal growth retardation and short stature. Craniofacial anomalies–short noses and anteverted nares were observed in 94.7% (18/19) of patients. Small hands and/or feet were present in 16 patients, skin manifestations (hirsutism or mottled skin) in six, and hearing loss in four. Genetic testing identified 19 variants in *NIPBL* (78.9%, 15/19), *SMC1A* (10.5%, 2/19), and *RAD21* (10.5%, 2/19), including 13 novel variants. *NIPBL* null variants correlated significantly with more severe growth impairments (*P* = 0.016) and microcephaly (*P* = 0.004). Although complete protein function loss often correlated with more severe clinical presentations, no significant difference in clinical scoring was observed (*P* = 0.600). Three patients treated with recombinant human growth hormone showed heterogeneous responses.

**Interpretation:**

This study highlights the clinical heterogeneity of CdLS and suggests a potential link between specific genetic variants and disease severity. These findings warrant further research to optimize treatments and better understand the functional impact of these genetic variants.

## Graphical abstract

*NIPBL* variants (78.9%) dominate 19 Chinese pediatric Cornelia de Lange syndrome (CdLS). Universal craniofacial anomalies (94.7%) and developmental delay (84.2%) were observed. *NIPBL* null variants are associated with severe growth impairment and microcephaly, yet overall clinical severity remains heterogeneous, underscoring genotype‐phenotype complexity

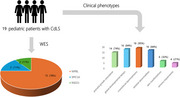

## INTRODUCTION

Cornelia de Lange syndrome (CdLS) is a rare genetic disorder characterized by a wide array of clinical manifestations that affect multiple organ systems.^1^ Hallmark features include distinct facial anomalies such as synophrys, short nose, anteverted nares, long and smooth philtrum, thin upper lip vermilion, and downturned corners of the mouth. Additional clinical signs often include growth retardation, microcephaly, developmental delays, cognitive impairment, hirsutism, and limb anomalies. CdLS often involves multiple systems, affecting cardiac, gastrointestinal, musculoskeletal, auditory, and urogenital structures.^2^, ^3^ Estimating the prevalence of CdLS is challenging due to the frequent underdiagnosis or lack of recognition of milder phenotypes. Published estimates of the prevalence range from approximately 1 in 100 000 to as high as 1 in 10 000. Data from the European Surveillance of Congenital Anomalies (EUROCAT) database suggest a prevalence of the classical form of CdLS at 1 in 50 000, while the milder form's incidence is likely underreported.^4^

CdLS is linked to abnormalities in the cohesion of complex components or their regulatory factors. Currently, seven genes associated with CdLS have been identified: *NIPBL* is the most prevalent, followed by *SMC1A*, *SMC3*, *RAD21*, *BRD4*, *HDAC8*, and *ANKRD11*.^5^, ^6^ Diagnosing CdLS involves a combination of clinical evaluation and genetic testing.^3^ Despite significant research efforts, there is still a lack of comprehensive reports on the clinical phenotypes and genetic spectrum of CdLS within the Chinese population.

This study aimed to broaden the genetic spectrum and elucidate genotype‐phenotype correlations by examining the clinical and genetic characteristics of 19 Chinese patients with CdLS. Additionally, by assessing the efficacy of recombinant human growth hormone (rh‐GH) therapy in three patients, we aimed to provide valuable insights and references for pediatricians in the diagnosis and management of this condition.

## METHODS

### Ethical approval

This study was approved by the Ethics Committee of Beijing Children's Hospital ([2021]‐E‐110‐Y). Informed consent for genetic analysis was obtained from the parents or guardians of all patients.

### Patients

This study included 19 unrelated children who were referred to Beijing Children's Hospital between September 2016 and August 2024. Detailed medical histories were recorded by pediatricians. The preliminary diagnosis of CdLS was established based on clinical manifestations and laboratory examinations, in accordance with the diagnostic criteria for CdLS by Kline et al.^2^, ^3^

The severity of the syndrome was assessed based on the scoring system proposed by the CDLS International Consensus Statement.^3^ Cardinal features (two points each) include synophrys, short nose, long and/or smooth philtrum, thin upper lip vermilion and/or downturned corners of the mouth, hand oligodactyly and/or adactyly, and congenital diaphragmatic hernia, and suggestive features (one point each) include global developmental delay and/or intellectual disability, prenatal growth retardation, postnatal growth retardation, microcephaly, small hands and/or feet, short fifth finger, and hirsutism.^3^ A total score of 11 points or more (with at least three cardinal features) indicates a classic CdLS. A score of 9 or 10 points (with at least two cardinal features) suggests a non‐classic CdLS. Furthermore, a score of 4–8 points (with at least one cardinal feature) necessitates molecular testing.^3^

### Genetic analysis

The genomic DNA of the parents and proband was extracted using a QIAamp Blood Midi Kit (QIAGEN). The DNBSEQ‐T7 sequencing platform (MGI) was used to perform whole exome sequencing, producing 150 base pair paired‐end reads. The clean reads were subsequently assembled and aligned using the MyGenostics next‐generation sequencing analysis platform. The coverage and sequencing quality of the target regions were thoroughly evaluated. Additionally, the Flash analysis platform was employed to assess the pathogenicity of the variants and to identify potential variant loci. The pathogenicity of genetic variants was assessed using the American College of Medical Genetics criteria and predicted using in silico methods and tools such as REVEL, SIFT, PolyPhen2, Mutation Taster, and GERP++. Sanger sequencing was performed with an ABI3730xl sequencer (Applied Biosystems) to validate the results and compare them with the sequencing data.

### Statistical analysis

Statistical analysis was conducted using IBM SPSS Statistics (22.0 Version). When the continuous variable follows a normal distribution, it is represented as the mean ± standard deviation; otherwise, it is represented as the median (interquartile range [IQR]). The Mann–Whitney *U* test or Student's *t‐*test was used to compare continuous variables, and Fisher's exact test was applied to compare categorical variables. A *P*‐value < 0.05 was deemed statistically significant.

## RESULTS

### Genetic characteristics

As shown in Table 1, three CdLS‐related genes were identified in 19 patients using whole‐exome sequencing. *NIPBL* variants were detected in 15 patients (78.9%), comprising six missense, three nonsense, two frame‐shift, and four splicing variants. Of these, six were previously reported, namely c.6242G>T (p.G2081V), c.133C>T (p.R45*), c.3316C>T (p.R1106*), c.5329‐15A>G, c.4829T>G (p.L1610R), and c.64+1G>A.^7^, ^8^, ^9^, ^10^, ^11^, ^12^ *SMC1A* variants were detected in two patients, including one missense and one splice variant. *RAD21* variants were identified in two additional patients, consisting of a frameshift variant and a large deletion variant. Both the *SMC1A* and *RAD21* variants are novel.

**TABLE 1:** Variants identified in 19 patients with Cornelia de Lange syndrome (CdLS)

Case	Sex	Gene	cDNA	Amino acid	Type	Status	Origin	CdLS score
1	F	*NIPBL*	c.6865G>C^†^	p.A2289P	Missense	Het	*de novo*	11
2	M	*NIPBL*	c.7379C>T^†^	p.S2460F	Missense	Het	*de novo*	10
3	F	*NIPBL*	c.6242G>T	p.G2081V	Missense	Het	*de novo*	11
4	M	*NIPBL*	c.6589+5G>T^†^	p.?	Splicing	Het	*de novo*	13
5	F	*NIPBL*	c.741C>G^†^	p.Y247*	Nonsense	Het	*de novo*	15
6	M	*NIPBL*	c.133C>T	p.R45*	Nonsense	Het	*de novo*	14
7	F	*NIPBL*	c.3626delT^†^	p.L1210Wfs*24	Frame‐shift	Het	*de novo*	15
8	F	*NIPBL*	c.8011_8012delGAinsTC^†^	p.E2671S	Missense	Het	*de novo*	9
9	M	*NIPBL*	c.4829T>G	p.L1610R	Missense	Het	*de novo*	10
10	M	*NIPBL*	c.64+1G>A	p.?	Splicing	Het	*de novo*	12
11	F	*NIPBL*	c.64+2_64+3insT^†^	p.?	Splicing	Het	*de novo*	12
12	M	*NIPBL*	c.8227T>C^†^	p.S2743P	Missense	Het	*de novo*	6
13	F	*NIPBL*	c.3316C>T	p.R1106*	Nonsense	Het	*de novo*	15
14	F	*NIPBL*	c.19_20insC^†^	p.His7profs*12	Frame‐shift	Het	*de novo*	10
15	M	*NIPBL*	c.5329‐15A>G	p.?	Splicing	Het	Maternal	13
16	M	*RAD21*	c.737delC^†^	p.P246Lfs*8	Frame‐shift	Het	*de novo*	5
17	M	*RAD21*	8q24.11q24.21^†^	‐	Large deletion	Het	*de novo*	12
18	M	*SMC1A*	c.2792A>G^†^	p.Q931R	Missense	Hemi	Maternal	8
19	M	*SMC1A*	c.2157‐5T>C^†^	p.?	Splicing	Hemi	Maternal	12

Abbreviations: CdLS, Cornelia de Lange syndrome; F, female; M, male; Het, heterozygous; Hemi, hemizygous.

^†^ The novel variants.

### Clinical characteristics

The cohort consisted of 11 males (57.9%) and 8 females (42.1%), with a mean age of 58 ± 46 months at diagnosis. The median height was −2.6 SDS (standard deviation score) (IQR: −4.2 to −1.9). Comprehensive clinical evaluations were conducted (Table 2). Twelve (63.1%) patients scored above 10 on the clinical feature scale, indicative of classic CdLS. Growth and developmental abnormalities were prevalent, with 16 (84.2%) exhibiting global developmental delay and 14 (73.7%) experiencing prenatal growth retardation and short stature. Craniofacial dysmorphisms were common, including thin upper lip vermilion/downturned mouth corners (100.0%, 19/19), short nose/anteverted nares (94.7%, 18/19), long/smooth philtrum (73.7%, 14/19), synophrys (68.4%, 13/19). Skeletal abnormalities were frequently observed, including small hand/feet (84.2%, 16/19), fifth finger clinodactyly (73.7%, 14/19), and thumb abduction (15.8%, 3/19). Only six patients exhibited hirsutism. Other noted anomalies included heart defects (31.6%, 6/19), hearing impairment (21.0%, 4/19), renal anomalies (15.8%, 3/19), myopia (10.5%, 2/19), gastrointestinal issues (5.3%, 1/19), and seizures (5.3%, 1/19). Additionally, among the 11 male patients, three had micropenis, two had hypospadias, and two had cryptorchidism.

**TABLE 2:** Clinical features in the cohort of 19 patients with Cornelia de Lange syndrome (CdLS)

Clinical features	*NIPBL* (*n* = 15)	*SMC1A* (*n* = 2)	*RAD21* (*n* = 2)
Sex (M/F)	7/8	2/0	2/0
Growth and developmental abnormalities			
Prenatal growth retardation	12	1	1
Short stature	12	1	1
Global developmental delay	13	2	1
Craniofacial dysmorphism			
Microcephaly	4	0	0
Synophrys	12	0	1
Short nose and/or anteverted nares	14	2	2
Long philtrum and/or smooth philtrum	13	1	0
Thin upper lip vermilion and/or downturned mouth corners	15	2	2
Ptosis	4	0	0
Skin manifestation			
Hirsutism	5	0	1
Mottled skin	5	0	1
Simian line	9	0	1
Skeletal abnormalities			
Small hand and/or feet	13	2	1
Short fifth finger	10	0	2
Fifth finger clinodactyly	12	0	2
Thumb abduction	2	0	1
2–3 toe syndactyly	1	0	0
Scoliosis	1	0	1
Pectus excavatum	3	0	1
Dislocation or dysplasia of the hip joint	1	0	0
Visual/Hearing abnormalities			
Myopia	2	0	0
Hearing impairment	2	1	1
Gastrointestinal anomalies			
Intestinal malrotation	0	0	1
Gastroesophageal reflux	0	0	1
Abnormalities of the external genitalia			
Micropenis	2	1	0
Hypospadias	1	1	0
Cryptorchidism	2	0	0
Other abnormalities			
Seizures	1	0	0.
Heart defect	4	1	1
Renal anomalies	1	0	2

Abbreviations: F, female; M, male.

### Genotype‐phenotype correlations in *NIPBL* gene

Patients with null variants (nonsense, frameshift, large deletions) in the *NIPBL* gene (Table 3) had significantly lower height SDS at diagnosis compared to those with other types of variants (−4.4 vs. −2.2, *P* = 0.016). Null variants were also strongly associated with microcephaly (*P* = 0.004), suggesting that complete loss‐of‐function mechanisms may contribute to more severe growth restriction and skeletal anomalies. However, other clinical features, including synophrys, craniofacial characteristics (e.g., short nose and long philtrum), global developmental delay, and prenatal/postnatal growth retardation, did not show statistically significant differences between the two groups (all *P* > 0.05). Additionally, clinical scoring did not reveal significant differences between the null and other variant groups (*P* = 0.600), although Figure S1 illustrates more pronounced facial dysmorphism and limb abnormalities in patients with null variants.

**TABLE 3:** Genotype‐phenotype correlations based on types of *NIPBL* variants

Variables	Null variants (*n* = 5)	Other variants (*n* = 10)	*P*‐value
Sex (M/F)	1/4	6/4	0.282
Age at diagnosis (months)	37 ± 27	77 ± 52	0.138
Height at diagnosis (SDS)	−4.4 ± 1.0	−2.2 ± 1.6	0.016
Clinical features^†^, *n* (%)			
Synophrys	4 (80.0)	8 (80.0)	1.000
Short nose and/or anteverted nares	5 (100.0)	9 (90.0)	1.000
Long philtrum and/or smooth philtrum	5 (100.0)	8 (80.0)	0.524
Thin upper lip vermilion and/or downturned corners of the mouth	5 (100.0)	10 (100.0)	‐
Global developmental delay (mild or severe)	4 (80.0)	2 (20.0)	0.089
Prenatal growth retardation	4 (80.0)	8 (80.0)	1.000
Postnatal growth retardation	5 (100.0)	7 (70.0)	0.505
Microcephaly	4 (80.0)	0	0.004
Small hands and/or feet	5 (100.0)	8 (80.0)	0.524
Short fifth finger	5 (100.0)	5 (50.0)	0.101
Hirsutism	3 (60.0)	2 (20.0)	0.251
Clinical score, *n* (%)			0.600
Classic type	4 (80.0)	6 (60.0)	
Non‐classic type	1 (20.0)	3 (30.0)	
Genetic identification	0	1 (10.0)	

Abbreviations: F, female; M, male; SDS, standard deviation score.

^†^ Features related to the Cornelia de Lange syndrome of clinical score. No patients in this cohort had hand oligodactyly or congenital diaphragmatic hernia.

### rh‐GH therapy

Three patients underwent rh‐GH therapy at the request of their parents. Patient 1, a girl diagnosed with CdLS at age 3, began rh‐GH treatment at age 5 with a dosage of 0.15–0.20 IU/kg/day. After 18 months, her height increased by 8 cm; however, her parents opted to discontinue the treatment, citing an inadequate response. Patient 9, a boy diagnosed at age 10, received rh‐GH therapy at 0.15 IU/kg/day during the first year, achieving a height increase of 12 cm, and he continues to receive the treatment. Patient 14, an 11‐year‐old girl who had yet to show signs of puberty, began rh‐GH treatment at age 7. Within 1 year, her height increased by 10 cm, although the specific dosage was not recorded. The therapy was eventually discontinued due to a significant enlargement of her hands and feet.

## DISCUSSION

This study provides a comprehensive analysis of the clinical and genetic characteristics of 19 pediatric patients with CdLS, underscoring its clinical complexity and genetic heterogeneity. Clinical manifestations of CdLS exhibit significant variability among individuals, with not all patients displaying typical features. While abnormalities of the facial and limb vary among patients, certain symptoms, including cleft palate, diaphragmatic hernia, congenital diaphragmatic hernia, absence of the upper arm or forearm, oligodactyly, anonychia, and radial head subluxation, were notably absent in this cohort.^3^, ^4^

Currently, seven genes have been associated with CdLS, with *NIPBL* being the most frequently implicated gene.^3^ In our study, three pathogenic genes were identified: *NIPBL, RAD21*, and *SMC1A. NIPBL* variants accounted for 78.9% of the cases, aligning with previous reports on CdLS.^13^ There exists a strong correlation between genotype and clinical phenotype in patients with CdLS. Specifically, those with nonsense or frameshift variants in the *NIPBL* gene exhibit the most severe clinical phenotypes, splicing variants are associated with moderate phenotypes, and missense variants correspond to milder phenotypes.^3^, ^14^ Based on a clinical scoring system, our study found that of the 15 patients with NIPBL variants, 10 exhibited the classic phenotype, four had the non‐classic phenotype, and one was classified based on genetic testing. We found that null variations (nonsense, frameshift, and large deletions) are likely associated with severe growth impairments and distinct skeletal phenotypes, such as microcephaly, due to complete functional disruption. However, broader clinical severity, as assessed by the classic/non‐classic scoring system for the *NIPBL* gene, seems independent of the variant type. Patients with null variants presented with more pronounced facial dysmorphism and limb abnormalities than those with other genotypes (Figure S1). Previously, it was thought that *NIPBL* variants led to more severe clinical manifestations than other gene variants.^14^ However, in this study, it was observed that patients with *NIPBL* missense mutations exhibited milder phenotypes, with less frequent occurrences of intellectual developmental abnormalities and epilepsy. These findings underscore the gene‐specific phenotypic effects of null variants, emphasizing the need for larger studies to explore the underlying molecular mechanisms.

Prenatal growth retardation and short stature were observed in 73.7% of cases, while 84.2% of the patients exhibited global developmental delay. These findings align with the reports by Dempsey et al.^15^ and Thellier et al.,^16^ which emphasize the significant growth and developmental challenges faced by patients with CdLS. The high incidence of these issues underscores the importance of early intervention and tailored growth monitoring in these individuals. In our cohort, the majority of patients exhibited craniofacial abnormalities, such as synophrys, short noses, and/or anteverted nares. These phenotypic manifestations are consistent with previously documented diagnostic criteria for CdLS in the clinical literature. A key craniofacial feature identified was the attenuated vermilion borders of the upper lip (100% prevalence), further supporting the syndromic facial gestalt in CdLS. Ophthalmic and auditory comorbidities were less prevalent, with myopia (10.5%) and sensorineural hearing loss (21.0%) being the principal findings, suggesting that these manifestations require systematic surveillance due to their prognostic implications.^17^ Cardiovascular malformations (31.6%) and nephrological anomalies (15.8%) were identified as predominant visceral manifestations, highlighting the characteristic multisystem involvement in CdLS pathogenesis. This phenotypic constellation underscores the need for standardized surveillance protocols that include serial cardiological evaluations (echocardiography) and renal function monitoring (ultrasonography with glomerular filtration rate assessment), supported by coordinated multidisciplinary management.

Gastroesophageal reflux disease (GERD) is considered one of the common clinical symptoms of CdLS.^4^ Notably, gastrointestinal symptoms were observed in only one participant (Patient 17), who experienced recurrent vomiting due to a large deletion in the *RAD21* gene. Following a 24‐h pH monitoring, GERD was identified. Other patients did not undergo diagnostic testing due to the absence of gastrointestinal symptoms. The low detection rate of GERD in this study might be attributed to the small sample size and the initial assessment conducted in the endocrine department, where the primary focus was on endocrine disorders.

Diagnoses of CdLS are predominantly established postnatally, with physicians relying on the characteristic clinical features in conjunction with genetic testing. However, the timing of diagnosis may vary due to phenotypic differences among individuals. In this study, the mean age at diagnosis was 58 ± 46 months, with most diagnoses occurring in early childhood. No significant correlation was found between clinical symptoms and age at diagnosis, suggesting that some children may have received a delayed diagnosis due to limited physician experience. Notably, patient 15, who had an *NIPBL* variant inherited from his mother, displayed mild symptoms, such as synophrys and excessive back hair. The mother had arched eyebrows, a slightly elongated philtrum, self‐awareness learning difficulties since childhood, hirsutism, short stature, and small hands and feet. This suggests the possibility of a mild CdLS phenotype under the same genetic background. In contrast, other patients with *NIPBL* variants were identified as having sporadic variants, consistent with the autosomal dominant inheritance pattern.

Once diagnosed, patients with CdLS can benefit from appropriate rehabilitation therapy, rh‐GH treatment, and surgical correction for skeletal deformities. Here we evaluated the efficacy of rh‐GH therapy in three patients. The administration of rh‐GH led to varying responses among patients, with height increases ranging from 8 to 12 cm over periods of 12–18 months. This aligns with previous reports in the literature indicating that patients experienced a height increase of 1.1 SDS following rh‐GH treatment, along with significant improvements in the Gesell developmental assessment and no adverse effects.^18^ However, a study by Li et al.^13^ reported one patient developing elevated blood glucose levels of 43.16 mmol/L post‐treatment; blood glucose levels normalized after stopping rh‐GH, with concurrent insulin and metformin treatment. These cases illustrate varying responses to rh‐GH therapy, which may be influenced by genetic and phenotypic characteristics. These outcomes highlight the importance of individualized monitoring and careful dosage assessment to optimize treatment effectiveness and minimize adverse effects. While rh‐GH treatment shows promise, its use should be approached cautiously to minimize the risks and maximize the benefits. Future research should focus on supplementing the data on the benefits of rh‐GH therapy through long‐term follow‐up studies.

In conclusion, this study analyzed 19 Chinese cases of CdLS associated with variants in the *NIPBL*, *SMC1A*, and *RAD21* genes, identifying 13 novel variants. *NIPBL* variants were the most prevalent in our cohort. The clinical manifestations observed in these patients were consistent with those reported in other ethnic groups, although there were differences in the frequency of certain features. The study's limitations include potential selection bias, as it was conducted at a single center, highlighting the need for more comprehensive research across diverse populations. Although these findings provide valuable insights into CdLS, caution should be exercised when interpreting the functional significance of these variants. Future research should aim to expand upon these findings to enhance the understanding and improve clinical management strategies.

## CONFLICT OF INTEREST

The authors declare no conflict of interest.

## Supporting information



Supporting Information
