# Supplementary material for: Clinical and genetic characteristics of Cornelia de Lange syndrome in pediatric patients
Source: Pediatr Investig. 2025 Jul 2;9(3):293–9. doi: 10.1002/ped4.70013 (PMC12442446; doi:10.1002/ped4.70013)
Supplement: Supplementary file 1 — Supporting Information [file PED4-9-293-s001.pdf]

**Supplementary Material for**

# Clinical and genetic characteristics of Cornelia de Lange syndrome in pediatric patients

Xiaoqiao Li, Ming Cheng, Min Liu, Wenjing Li, Yuchuan Li, Bingyan Cao, Liya Wei, Yuan Ding, Xi Meng, Lele Li, Chunxiu Gong

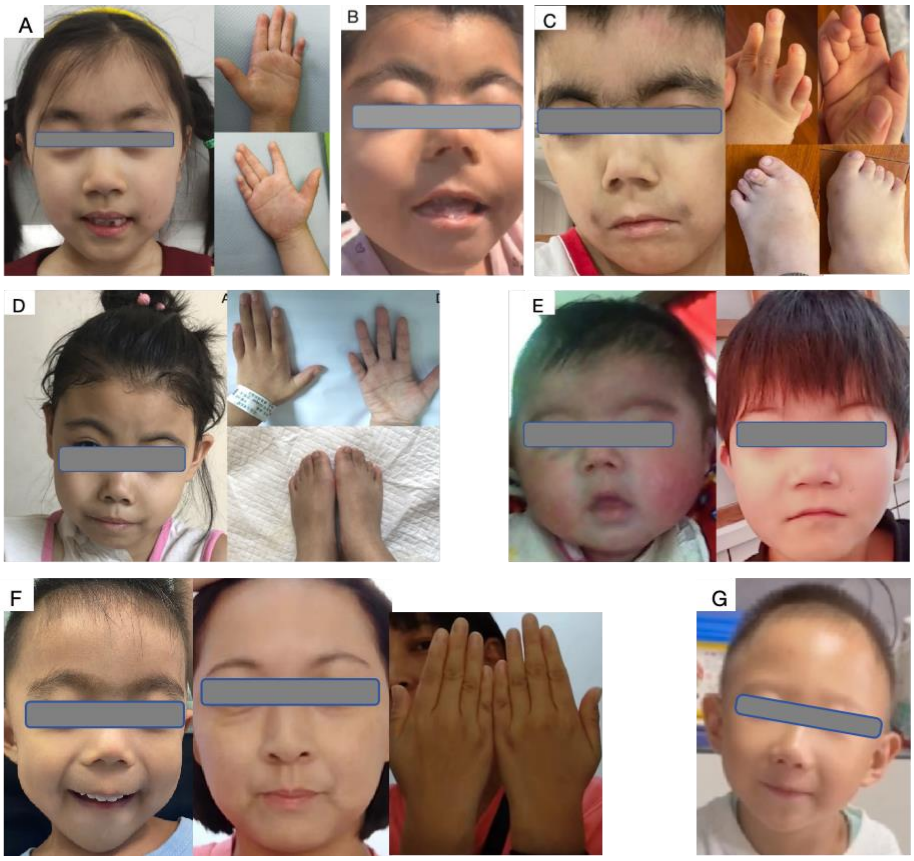

**Figure S1**. Clinical manifestations of some patients. (A) Patient 1: The patient presents with short stature, mild intellectual disability, short and curved fifth fingers, and a right single transverse palmar crease. Following 18 months of rh-GH therapy, she experienced an 8 cm increase in height; however, the treatment was deemed ineffective and subsequently discontinued. (B) Patient 5: The patient exhibits typical facial features of CdLS, ptosis, severe developmental delay, intellectual disability, and hearing impairment. (C) Patient 7: This patient has the classical facial appearance of CdLS, hirsutism, severe intellectual disability, short fifth fingers, and short, malformed toes. (D) Patient 11: The patient has moderate intellectual disability, short fifth fingers with a single transverse palmar crease, and syndactyly of the second and third toes. (E) Patient 14: The patient has mild intellectual disability. The images show the patient at ages 1 and 11 years. She received rh-GH treatment at the ages of 7, resulting in a 10 cm growth spurt; however, the treatment was discontinued due to significant enlargement of her hands and feet noted by the parents. (F) Patient 15: The patient's intellectual capacity is normal, with the genetic variant being inherited from the mother. The image on the right shows the patient's mother, who has arched eyebrows, a slightly elongated philtrum, self-awareness learning difficulties since childhood, hirsutism, and short stature (height of 152 cm), along with

small hands and feet. (G) Patient 19: This patient does not have synophrys but displays mild anteverted nostrils, a thin upper lip, hypospadias, and mild intellectual disability.
